# Transverse colon perforation in the mediastinum after esophagectomy: a case report

**DOI:** 10.1186/s40792-020-00862-5

**Published:** 2020-05-25

**Authors:** Takuro Konno-Kumagai, Tadashi Sakurai, Yusuke Taniyama, Chiaki Sato, Kai Takaya, Ken Ito, Takashi Kamei

**Affiliations:** Division of Advanced Surgical Science and Technology, Graduate School of Medicine, University of Tohoku, 1-1 Seiryo-machi, Aoba-ku, Sendai, Miyagi 980-8574 Japan

**Keywords:** Hiatal hernia, Post-esophagectomy, Perforated colon, Septic shock

## Abstract

**Background:**

While anastomotic leakage, recurrent laryngeal nerve paralysis, and pneumonia are well-known complications of esophagectomy, the incidence of hiatal hernia after esophagectomy for carcinoma has been reported to only be between 0.6 and 10%. We report a very rare case of hiatal hernia with transverse colon rupture in the mediastinum after esophagectomy in a 65-year-old woman.

**Case presentation:**

The patient underwent definitive chemoradiotherapy for clinical stage IIA esophageal squamous cell carcinoma and salvage esophagectomy with gastric tube reconstruction through a posterior mediastinum route for residual carcinoma. Three years after the initial surgery, two metastatic nodules in the lateral and posterior segments of the liver were detected on follow-up CT and were treated with oral anticancer drugs. After 6 months, the patient was readmitted for anorexia. Upon admission, computed tomography revealed an ileus caused by a hiatal hernia. Emergent operative repair was performed; an incarcerated herniation of the transverse colon was perforated in the mediastinum, and partial transverse colon resection and colostomy were performed. Intensive care was required to control septic shock after surgery, and the patient was discharged on the 53rd postoperative day.

**Conclusions:**

Cases of hiatal hernia with digestive tract prolapsing into the mediastinum after esophagectomy with reconstruction through posterior mediastinum are rare but potentially life-threatening complications.

## Background

As esophagectomy for carcinoma of the esophagus becomes safer and less invasive thanks to improvements in medical treatment techniques and intensive care, patients survive longer with a lower incidence of complications. While respiratory complications and anastomotic leakage following esophagectomy are relatively common complications, diaphragmatic herniation is rare; the incidence of hiatal hernia after esophagectomy has been reported to be between 0.6 and 10% [1, 2]. Nevertheless, there are increasing reports of hiatal herniation following esophagectomy using minimally invasive techniques or neoadjuvant oncological therapies that have recently become the standard treatment for esophageal carcinoma and associated with the risk factors of postoperative hiatal hernia [1, 3, 4].

Surgical repair is often performed for symptomatic patients with hiatal hernias, and prophylactic repair is also performed for some asymptomatic patients, but not for all. In patients observed without surgical repair, emergent surgery is sometimes required because of bowel ischemia or strangulation and the postoperative course can be severe. Here, we report the successful management of a rare case of hiatal hernia involving a prolapse of the transverse colon ruptured in the mediastinum after esophagectomy.

## Case presentation

A 65-year-old Japanese woman was admitted to our hospital with dysphagia and was diagnosed with clinical T2N0M0 stage IIA [Union for International Cancer Control (UICC), 8th edition] esophageal squamous cell carcinoma. She was treated with concurrent chemoradiotherapy (CRT). The chemoradiotherapy protocol was consistent with that of the Japan Clinical Oncology Group trial 9906; the patient received two cycles of intravenous cisplatin infusions with continuous 5-fluorouracil (5-FU) infusion and concurrent radiotherapy of 60 Gy (30 fractions of 2 Gy) [5]. Two months after the entire course of CRT, esophagoscopy revealed a residual tumor and salvage esophagectomy was performed. Right thoracoscopic access for esophagectomy was achieved in the prone position, and hand-assisted laparoscopic technique was performed in the supine position. After the hiatus was dilated by manual blunt force through four fingerbreadths, gastric reconstruction was performed through the posterior mediastinal route and by cervical esophagogastric anastomosis. The gastric conduit was fixed to the hiatus with two non-absorbable sutures. The postoperative course was uneventful, and the patient was discharged on the 21st postoperative day and diagnosed with pathological stage IB (T1bN0M0) squamous cell carcinoma. Three years after esophagectomy, two metastatic nodules in the lateral and posterior segments of the liver were detected on follow-up CT. As the postoperative performance status of the patient had worsened, she was then treated with only oral anticancer drugs (tegafur-gimeracil-oteracil potassium).

**Fig. 1:**
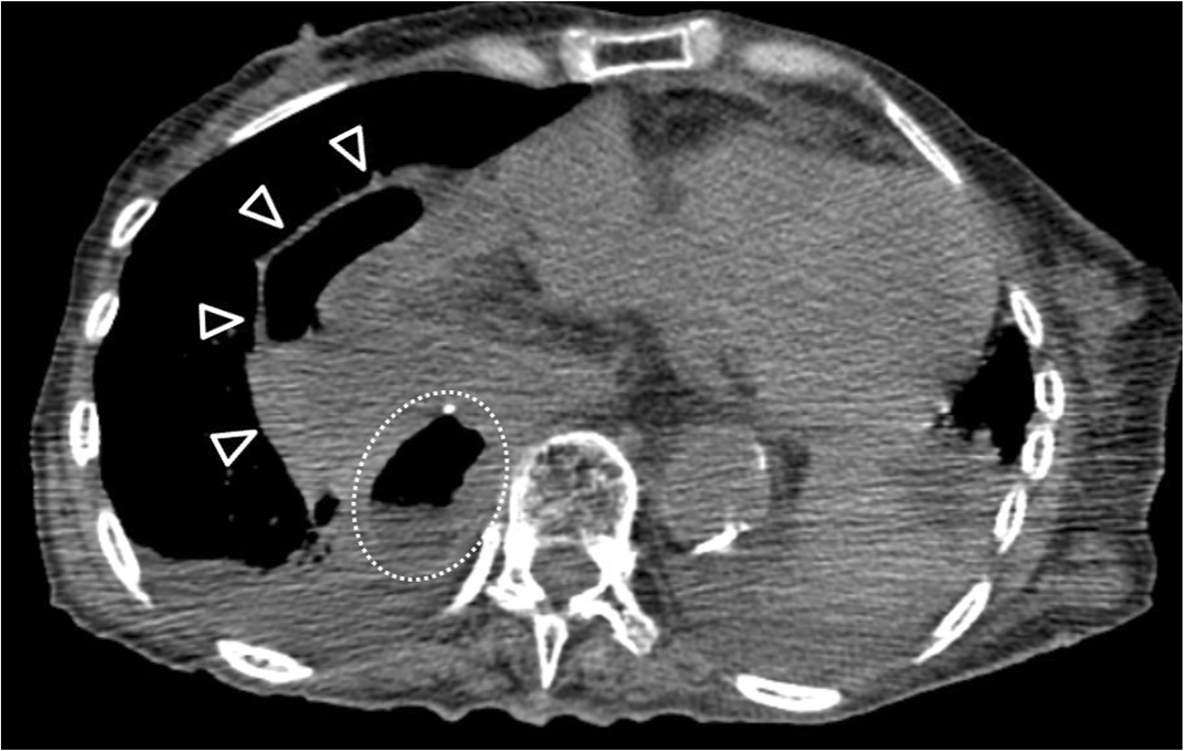
Computed tomography images. Non-contrast computed tomography revealing digestive tract prolapse into the right side of the mediastinum, which is distended with air and fluid (arrow heads). A moderate amount of pleural effusion is collected in both of the thoracic cavities. The gastric conduit is indicated in the circle

**Fig. 2:**
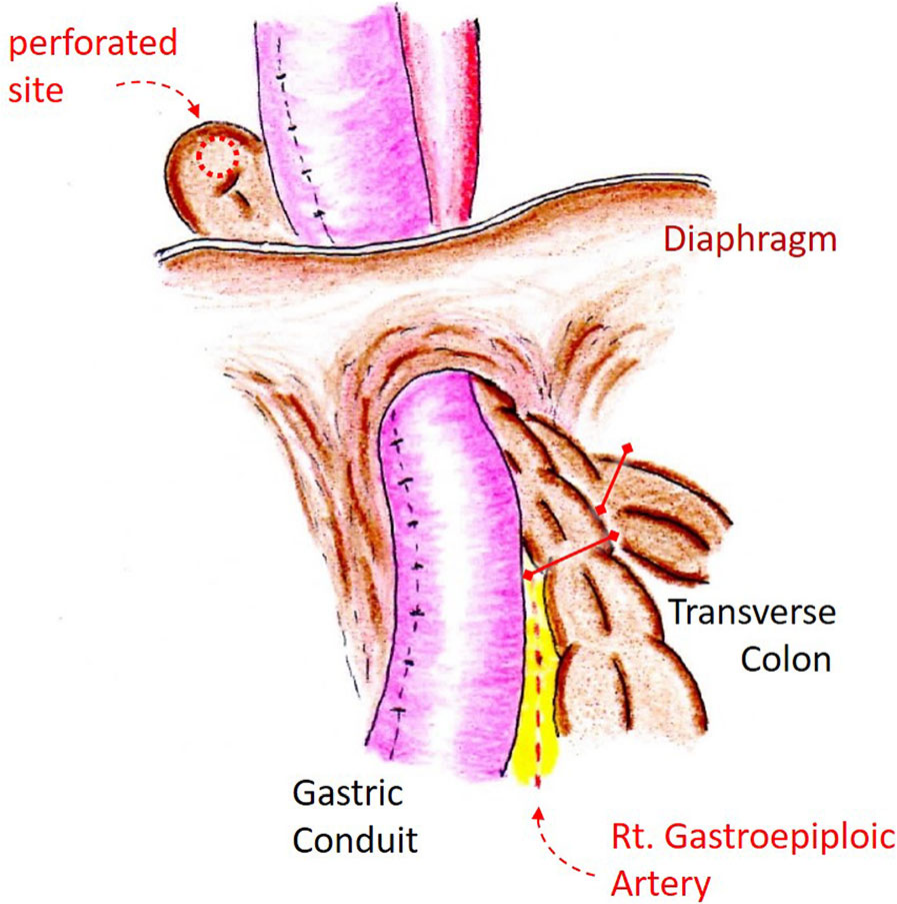
Schema of operative findings. This schema shows the prolapse of the transverse colon through the left side of diaphragmatic hiatal defect into the mediastinum around the back of the gastric conduit. Incarcerated herniation of the transverse colon was perforated in the mediastinum and was resected, as seen by the red lines. The liver was omitted in this schema

**Fig. 3:**
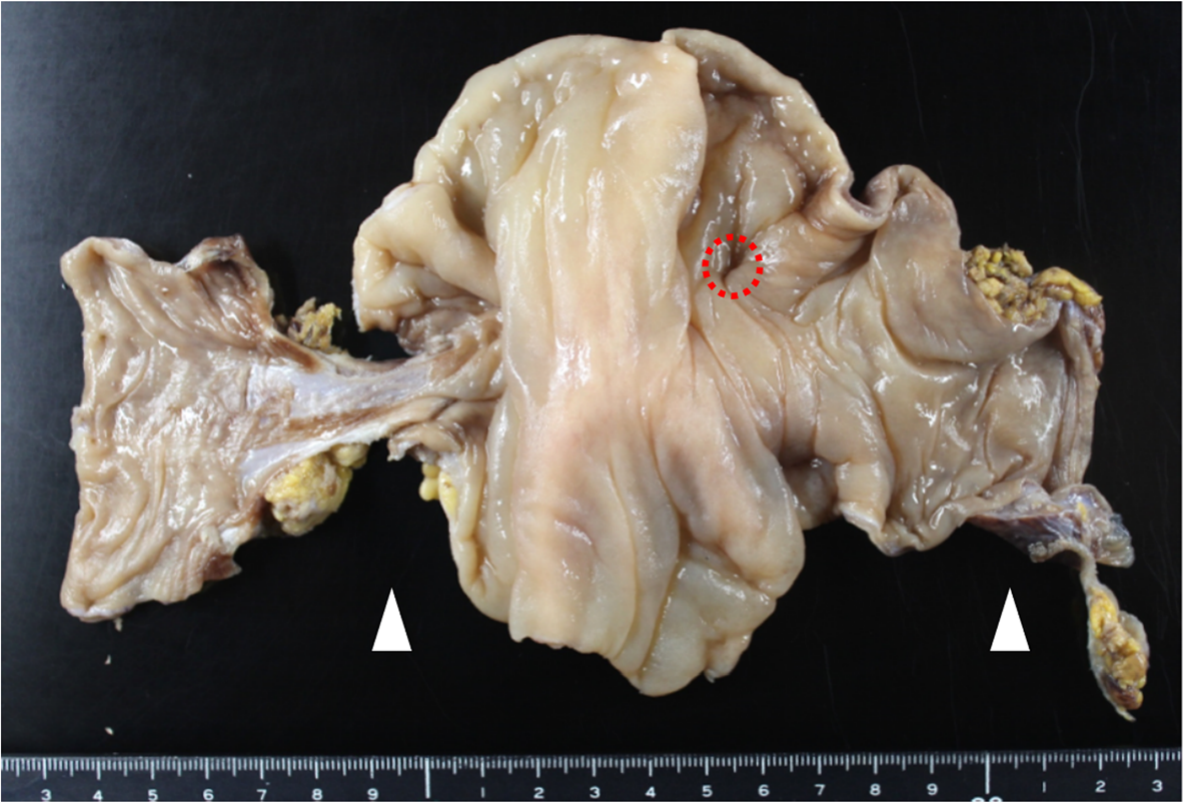
Macroscopic view of the resected specimen showing perforation of the colon (red circle) and the edematous colonic wall without necrosis between the constrictions (arrow heads)

Six months later, the patient was readmitted for anorexia of a few weeks’ duration. On admission, heart rate and mean blood pressure were 98/min and 59 mmHg, respectively, suggesting a state of shock. Peripheral blood examination showed that the white blood cells were in the normal range; however, the C-reactive protein (CRP) level was elevated to 32.1 mg/dL. Arterial blood gas analysis on room air showed acidosis. Blood urea nitrogen and creatinine levels were elevated to 91 mg/dL and 4.99 mg/dL, respectively, suggesting renal dysfunction or severe dehydration (Table 1). Non-contrast computed tomography revealed digestive tract prolapsing into the right side of the mediastinum, which was distended with air and fluid (Fig. 1). The patient was diagnosed with postoperative hiatal hernia with incarcerated digestive tract, and emergency operative repair was performed with open surgery through the abdomen. The distal transverse colon was incarcerated through the left side of diaphragmatic hiatal defect into the right mediastinum around the back of the gastric conduit (Fig. 2). When a part of the adhesion around the hiatus was exposed, digestive fluid was eluted from the mediastinum. It was difficult to expose the colon safely due to the strength of the adhesion in the mediastinum. In addition, the gastroepiploic artery, preserved as the feeding artery and located on the left side of the gastric conduit, made it difficult to pull the transverse colon and expose the adhesion in the mediastinum. Finally, after manual repositioning of the herniated content, the transverse colon, incarcerated into the mediastinum, was resected due to perforation and a transverse colostomy was performed. After reduction of the herniated bowels, the mediastinum and abdominal cavity were manually lavaged and the drainage tube was placed in the mediastinum via the hiatus. The right side of the pleural cavity was also lavaged through the widened hiatus, without thoracotomy, which would have been invasive for the patient. The dilated diaphragmatic hiatal defect was closed with sutures, and the gastric conduit was fixed to the crus with 2–0 non-absorbable sutures. The total operation time was 233 min, and the intraoperative blood loss was 770 mL. Macroscopic analysis of the resected specimen revealed perforation in the edematous lesion of the colon without necrosis (Fig. 3). Postoperatively, the patient was transferred to the intensive care unit (ICU) and was on a ventilator. The patient was diagnosed with septic shock due to acute mediastinitis, and broad-spectrum antibiotics and vasopressor agents were administered. Continuous hemodiafiltration and polymyxin B hemoperfusion were administered for the first 3 days. The patient stayed in the ICU for 16 days, and oral intake was initiated on the 18th postoperative day. The patient was discharged from the hospital on the 53rd postoperative day. The patient’s condition deteriorated following surgery, and she did not receive any further chemotherapy or radiotherapy. Only palliative care services were provided, and she is alive one and half years after surgery.

**Table 1:** Laboratory findings on admission

Peripheral blood		
WBC	6100	/μl
RBC	232	10^4^/μl
Hb	8.0	g/dl
Hct	22.9	%
Plt	218	10^3^/μl
Biochemistry		
TP	5.1	g/dl
Alb	2.2	g/dl
T-bil	0.3	mg/dl
AST	28	U/l
ALT	6	U/l
ALP	256	U/l
γ-GTP	19	U/l
Na	139	mEq/l
K	2.5	mEq/l
Cl	99	mEq/l
BUN	91	mg/dl
Cre	4.99	mg/dl
Glucose	99	mg/dl
CRP	32.1	mg/dl
Coagulation		
PT	80.0	%
APTT	35.9	sec
Tumor marker		
CEA	6.1	ng/ml
SCC	1.3	ng/ml
Arterial Blood Gas Analysis (room air)		
pH	7.416	
pCO2	28.3	mmHg
pO2	92.6	mmHg
Lac	1.0	mmol/L
BE	-5.4	mmol/L

## Discussion

To the best of our knowledge, this is the first case report of transverse colon prolapsing through the hiatus into the mediastinum and rupturing, following an esophagectomy. According to previous reports, the contents of a hernia can vary, including structures such as the colon and/or small intestine, although it is not clear which organs have a greater risk of herniation through the hiatus. Unfortunately, the prolapse and rupture of the transverse colon into the mediastinum could result in a more severe postoperative course.

The incidence rate of postoperative diaphragmatic hernias after esophagectomy is estimated to be between 0.6 and 10% [1, 6]. This rate may vary depending on the operative technique, such as reconstruction route or postoperative follow-up time. When the retrosternal route is used, the hiatus is often closed tightly, and diaphragmatic herniation will occur less frequently [7]. On the other hand, in many Western countries, the mediastinal route is often used for adenocarcinoma of the esophagus. When the mediastinal route is selected, the diaphragmatic hiatus is manually widened in order to prevent obstruction of the gastric conduit. In our institution, the gastric conduit is fixed to the hiatus with two non-absorbable sutures routinely to avoid postoperative hiatal hernia. This procedure anchoring the stomach to the opened hiatus is recommended by Hamaloglu et al. [8]. However, others do not support the beneficial effect of such a fixation, and they argue this poses unnecessary risk to the primary vascular supply of the gastric conduit [9, 10]. Unfortunately, in our case, three and half years later, the transverse colon was prolapsed through the hiatus into the mediastinum despite fixation of the gastric conduit. Even when gastric conduit was fixed to the hiatus, hiatal hernia may occur because of the following reasons.

One possible hypothesis is intra-abdominal procedures causing postoperative hiatal hernia. Minimally invasive esophagectomies (MIE) are associated with a lower pulmonary complication rate, less abdominal pain, and shortened hospitalization period compared to an open procedure [1, 4, 9]; on the other hand, hiatal hernia following esophagectomy could be an increasing problem. Previous reports presumed that hiatal hernia occurs more often after MIE than open esophagectomy, because of fewer peritoneal adhesions in the hiatal region, resulting from minimal access techniques, such as thoracoscopic and laparoscopic surgery [11, 12]. Matthews et al. [1] reported that hiatal hernia after esophagectomy of the 506 cases occurred more frequently in laparoscopy than in open laparotomy. The incidence of postoperative hiatal hernia was 6.8–10.4% after laparoscopy and 1.8% after open laparotomy [1]. On the other hand, there was no difference in the incidence of postoperative hiatal hernia according to thoracic approaches. This result indicated that abdominal procedures could be more associated with hiatal hernia than intrathoracic procedures.

**Fig. 4:**
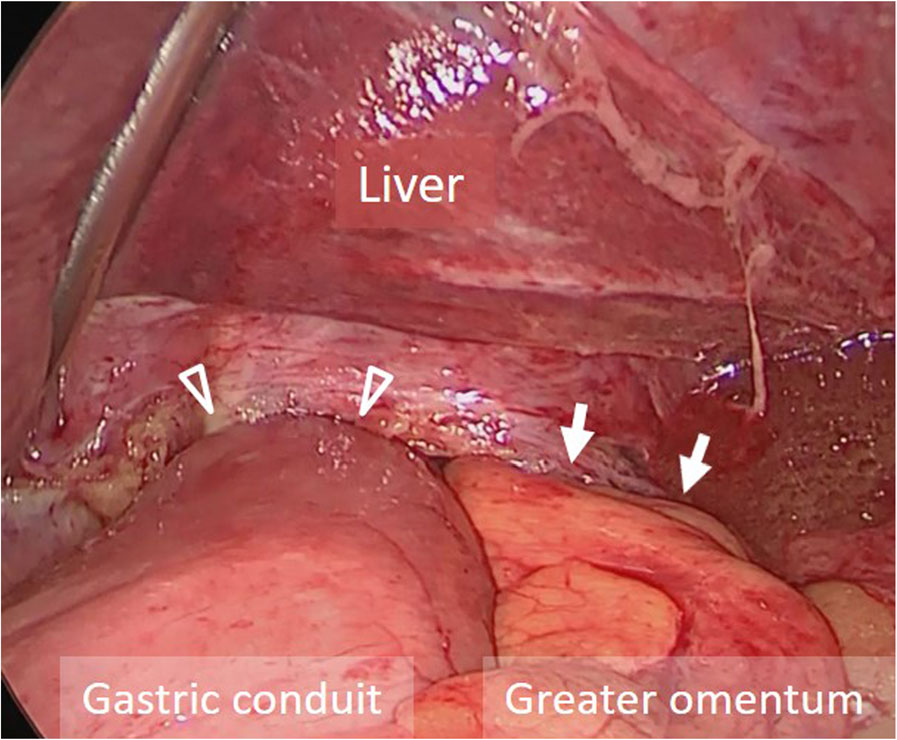
Another case of narrow gastric tube reconstruction in laparoscopic view. Greater omentum with right gastroepiploic artery (arrows) was located in the left side of gastric conduit (arrowheads)

Several studies have suggested that anatomical factors are also associated with the incidence of postoperative hiatal hernia [1, 4]. As Fig. 4 shows (the other reconstruction case), the liver is located on the right side of the abdomen and the gastric conduit could be fixed safely to the right side of the hiatus; this could prevent the digestive tract from prolapsing into the mediastinum through the right side of the orifice. On the other hand, because the gastric omentum, including the right epigastric artery, is located on the left side of the gastric conduit, preventing postoperative hiatal hernia through the left side could be insufficient. This theory coincides with previous reports indicating predisposing factors for left-sided predominance [1, 4].

Another study suggested that tissue fragility after neoadjuvant chemoradiation could be strongly related to risk factors for postoperative hiatal hernia [3, 7]. Late diaphragmatic hernias are associated with progressive hiatal dilatation secondary to diaphragm weakness or increased intra-abdominal pressure caused by a gibbus deformity [3, 7, 13]. These hypotheses relate to the current case, whose period of postoperative hiatal hernia was three and half years after the salvage operation following definitive chemoradiotherapy. Moreover, malnutrition caused by chemotherapeutic treatment for liver metastasis could be another risk factor for postoperative hiatal hernia in this case. Body weight loss after esophagectomy and chemotherapy causes the decrease of mesenteric fat, which could cause colon elongation and lead to increased colon mobility.

**Fig. 5:**
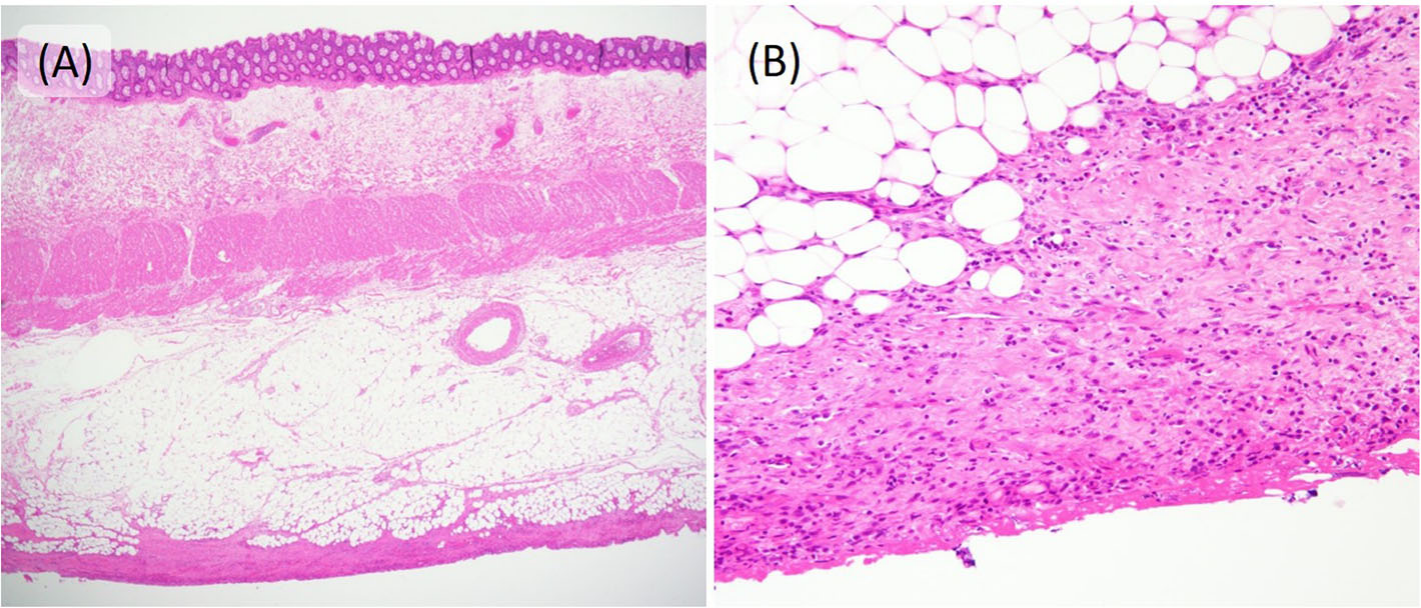
Histopathological findings near the site of the perforated transverse colon. **a** Edematous change is mainly observed in the submucosal layer, but there was no necrosis. **b** Inflammatory cell infiltration, including neutrophil and deposition of fibrin, is observed on the serosal membrane (hematoxylin-eosin staining, **a** × 20 magnification. **b** × 200 magnification)

Previously, Krause et al. [14] reported that hiatal hernia following esophagectomy and the transverse colon migrating into the hernia became ischemic and necrotic. In our case, however, necrotic lesion of the colon was not observed macroscopically. Pathological examination revealed edematous change of the resected colon specimens and inflammatory cell infiltration, including neutrophils and fibrin deposition, mainly on the serosal membrane (Fig. 5). It was therefore determined that the transverse colon in the mediastinum had already perforated a few hours earlier, caused by the elevation of intestinal inner pressure. According to Shinkawa et al. [15], colorectal perforation in the abdomen could be a life-threatening condition requiring emergency surgical intervention; despite recent advances in surgical techniques and intensive care, 17.5% of patients die during hospitalization. Colorectal perforation in the mediastinum, such as in this case, can cause acute respiratory distress syndrome and/or serious arrhythmias and may be more severe than when they occur in the abdominal cavity. The Acute Physiology and Chronic Health Evaluation (APACHE) II score [16] before surgery was 22; patients with APACHE II scores of 21–30 are estimated to have a mortality rate of 61% [17].

Symptomatic patients are usually treated immediately after diagnosis to prevent life-threatening complications. By contrast, there is no consensus regarding the treatment of asymptomatic patients who are often diagnosed incidentally during cancer surveillance imaging. Prophylactic repair in patients with hiatal hernia should be individualized according to their life expectancy, history of pre-existing disease, and the risk of the operation. Even if severe incarcerated hiatal hernia was found in patients after esophagectomy with reconstruction through a posterior mediastinal route, there is a possibility of saving severe cases with intensive care, as in the present case. Therefore, immediate diagnosis and surgical repair are required.

## Conclusions

This is the first case report of a hiatal hernia involving prolapse of the transverse colon ruptured after esophagectomy. As numbers of patients with esophageal carcinoma treated with neoadjuvant CRT or MIE increase, more patients could experience postoperative hiatal hernia. It should be remembered that postoperative hiatal hernia could be a life-threatening situation and immediate diagnosis and treatment are required.

## Data Availability

Data sharing is not applicable to this article as no datasets were generated or analyzed during the current study.

## Abbreviations

CT: Computed tomography
